# Novel Advances in the Role of Selective Estrogen Receptor Modulators in Hormonal Replacement Therapy: A Paradigm Shift

**DOI:** 10.7759/cureus.49079

**Published:** 2023-11-19

**Authors:** Gunjan Motlani, Vidhi Motlani, Neema Acharya, Apoorva Dave, Soumya Pamnani, Drishti Somyani, Shruti Agrawal

**Affiliations:** 1 Medicine, Jawaharlal Nehru Medical College, Datta Meghe Institute of Higher Education and Research, Wardha, IND; 2 Obstetrics and Gynecology, Jawaharlal Nehru Medical College, Datta Meghe Institute of Higher Education and Research, Wardha, IND

**Keywords:** hot flushes, vulvovaginal atropy, osteoporosis, hormonal replacement therapy, selective estrogen receptor modulators

## Abstract

Estrogen is a key regulatory hormone in the functioning of a female reproductive system. Estrogen hormone regulates many complex physiological processes, which has its role in reproduction and skeletal and cardiovascular systems by acting on estrogen receptors alpha (ERα) and beta (ERβ), which are nuclear transcription factors. Selective estrogen receptor modulators (SERMs) are now being used to treat bone loss, breast carcinoma, and menopausal symptoms, metabolic neurodegenerative because of their characteristics that allow them to function as both estrogen agonists and antagonists, depending on the target tissue. First-generation SERMs, such as Tamoxifen, are used in the management protocol for breast cancer, which is estrogen receptor (ER-positive). Raloxifene is a second-generation SERM that is a valuable adjunct used to treat and prevent osteoporosis in postmenopausal women and prevent compression fractures of the vertebral column. Novel SERM molecules are on the horizon, proven more potent and efficacious in preventing and treating osteoporosis. These include Ospemifene, lasofoxifene, bazedoxifene and arzoxifene. The benefits of Raloxifene versus that of Bazedoxifene are under trial. Despite their therapeutic benefits and actions, these medications are not without adverse effects, such as thromboembolic disorders. Increased risk of uterine cancer has been linked to Tamoxifen. This article delves into the world of SERMs, including their development and discovery. The newer SERMs in late development, ospemifene, lasofoxifene, bazedoxifene, and arzoxifene, are described in detail.

## Introduction and background

The therapeutic applications of estrogens and other hormones have attracted much attention in reproductive science over the past ten or more years [1]. Many hormonal changes take place as women approach menopause. The sharp decrease in circulating levels of 17 Beta-estradiol and estrone is the most prominent of these alterations. Progesterone level declines, and gonadotrophin (LH and FSH) secreted by the pituitary increases. The loss of cyclicity in menstruation and a drop in the estrogen level cause several symptoms (such as mood fluctuations, hot flashes, urogenital symptoms, vulvovaginal atrophy (VVA) and sleep disturbances) [2]. Long-term estrogen depletion has also been linked to (or related to) significant chronic disorders like osteoporosis, coronary artery disease, stroke, changes in lipid profiles, decreased insulin sensitivity, Alzheimer's disease, dementia, and breast cancer [3]. As postmenopausal symptoms may affect quality of life, certain physicians hesitate to start or continue therapy due to safety concerns. Menopause symptoms can be managed by hormonal and non-hormonal methods [4]. Menopausal hormone replacement therapy (HRT) is recommended for use in younger women who have symptoms and are under 60 years and within ten years of their last menstrual period. These recommendations are outlined in various guidelines and consistently indicate that the potential advantages of hormonal therapy generally outweigh the risks [5]. Observational epidemiological research studies strongly support the therapeutic use of steroid hormones, especially estrogen, in postmenopausal women for the long-term avoidance of illnesses and the short-term management of complaints like hot flashes. The long-term use of estrogens has additionally been associated with dangers like an increased chance of venous thromboembolism (VTE) and a higher risk of cancer of the endometrium, which can both be prevented by giving appropriate doses of progesterone in a woman with a healthy uterus [1].

## Review

Methodology

**Figure 1:**
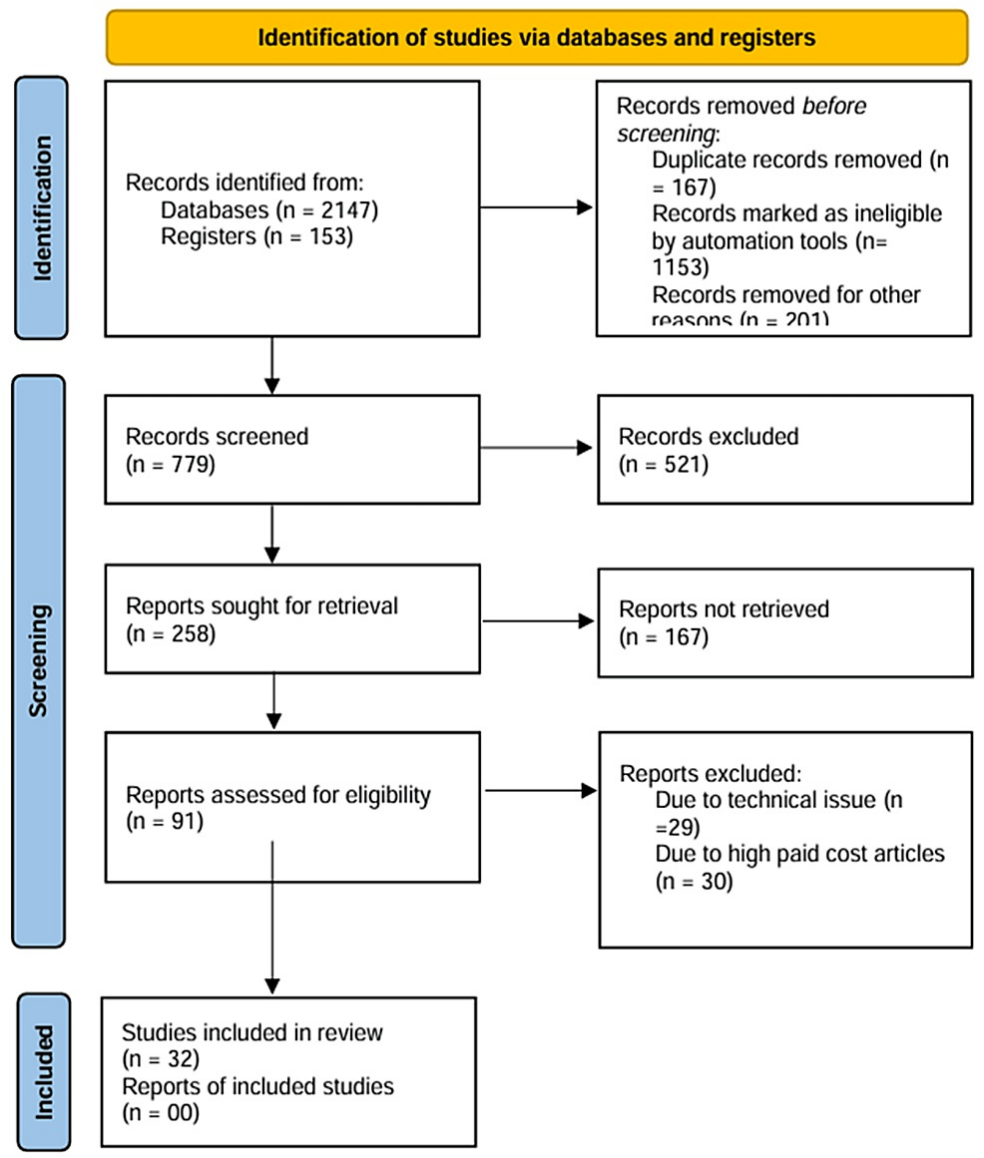
PRISMA flow diagram for literature search Adapted from PRISMA: Preferred Reporting Items for Systematic Reviews and Meta-Analyses

The recent advances in the treatment of selective estrogen receptor modulators (SERMs) and the new drugs were thoroughly reviewed using a literature search. A systematic search was undertaken through PubMed in September 2023 using the keywords “selective estrogen receptor modulators,” “hormonal replacement therapy,” and “recent advances.” We also looked for critical references of relevant studies from their respective bibliographies. Reference lists of pertinent publications and review papers were manually examined in addition to electronic database searches to find more studies. The selection process for the research that satisfied the inclusion criteria included observational studies, experimental studies, systematic reviews and meta-analyses that looked at the relationship between drugs like tamoxifen, raloxifene and their related outcomes. The inclusion of only peer-reviewed and published articles was taken into consideration. Two reviewers separately checked titles, abstracts, and full texts of the retrieved papers to see if they met the inclusion criteria before including them, and any inconsistencies were settled by discussion and agreement. The steps for inclusion studies are depicted in Figure 1.

Estrogen receptor (ER)

Estrogen signaling is a sophisticated and crucial biological mechanism that regulates neural and reproductive processes and the cardiovascular system. Estrogens impact cellular functions by interacting with the receptors for estrogen, which can be present in the nucleus, cytoplasm, or plasma membrane. There are two ER isoforms, ER alpha and ER beta, and they differ in their distribution patterns across tissues and regulation of transcription [6]. Numerous studies have demonstrated that growth factors (such as insulin-like growth factor (IGF), epidermal growth factor (EGF), and others), other substances, such as dopamine, and cAMP can promote ER activity and change the agonist/antagonist balance of SERMs. An increase in protein kinase A (a signal route) activity Particularly decreased the antagonistic potency of this and other SERMs while increasing the agonistic action of tamoxifen-like antiestrogens [7]. It is now recognized that estrogen pharmacology depends on additional factors besides the ERs and ligands, such as coregulatory proteins and gene promoter elements (GPE). The ERs have the property to bind with DNA response elements (DRE) through direct interaction or connecting to additional transcription factors that bind DNA, thus resulting in interactions with target genes. With all of these things considered, there seem to be various combinatorial options for establishing gene-specific and tissue regulation of SERMs [8]. The decrease in the levels of estrogen or loss of its receptors, as well as a more significant activity of ERs, are associated with the pathogenesis of various diseases such as cancers (endometrial cancer, Breast cancer, colon cancer, ovarian tumor, and cancer of prostate), neurodegenerative illnesses (Alzheimer's disease, stroke, multiple sclerosis, Parkinson's disease, dementia, etc.), osteoporosis, obesity, and heart diseases. Estrogens have been therapeutically employed as HRT for menopause, contraception, and infertility treatment [6].

Estrogens are essential for the growth, development, cognition, reproduction, maintenance of the skeleton, and other processes [9]. It is known that the ER-ligand complex controls gene transcription by interacting with a DRE. Accordingly, it has been hypothesized that particular configurations of the receptor-substrate complex influence distinct subsets of estrogen-responsive genes, leading to differential regulation that eventually results in tissue-selective consequences [10]. As a result of these interactions, pathologies such as malignancy (including breast, endometrial, colorectal, breast, and prostate), circulatory and metabolic disorders, and cognitive disorders including Alzheimer's and osteoporosis, can occur. The hormone estrogen has been used successfully in medical settings for infertility treatment, as menopause hormone therapy and oral contraception [9].

Selective ER modulator (SERM)

A wide range of nonsteroidal substances known as SERMs act as agonists for some target tissues and antagonists for other receptors of estrogen (ERs) [9,11].

Potential Benefits

Relief of vasomotor instability: Relief of vasomotor symptoms, sometimes known as hot flushes, is among the main reasons women start ERT. A hot flush is an extreme feeling of warmth that typically begins in the forehead, neck, top of the chest, or back but can affect the entire body. The duration of a hot flush might range from 30 to 5 minutes. Although the reason is unknown, alterations in the hypothalamus thermoregulatory center may be the source of these episodes. Hot flushes may happen due to a disruption in the thermoregulatory center brought on by the drop in estrogen levels, which causes dilatation of vessels and increased sweating. Hot flushes can occur in women as infrequently as just a couple times per year or even as frequently as multiple times daily. Most often happening at night, flushes of heat can wake a woman out of her sleep. Hot flushes can result in sleeplessness, fatigue, nausea, vomiting, night sweats, diaphoresis, dizziness, headache, and palpitations for some. The best treatment for reducing these vasomotor symptoms of menopause is the replacement of estrogen for about five years and then gradually tapering down estrogen when vasomotor symptoms subside [12,13].

Relief of urogenital and vaginal atrophy (VA): The urogenital sinus gives rise to the female reproductive system and lower urinary tracts; receptors that respond to estrogen are abundant in these organs, including the bladder trigone, vagina, and vestibule [14]. Earlier in life, before menopause, high estrogen levels support appropriate glycogen levels in the vaginal epithelial tissue, supporting healthy tissue maintenance within the vagina. The amount of glycogen in the epithelium of the vagina is crucial in creating an environment that is acidic with a pH level that is low and high in lactobacilli. As estrogen levels fall throughout menopause, the lining of the vagina thins and turns more brittle, and the glycogen amount in the vaginal epithelial cells also declines. An increase in the likelihood of vaginal infections like vaginosis caused by bacteria results from the vaginal pH rising and becoming more alkaline. The epithelial layer of the vaginal epidermis is less elastic, and there is less lubrication in the vagina. The abovementioned changes increase the possibility of vaginal dryness and pain in postmenopausal women during sexual activity [15,16]. VA can be treated with several nonhormonal methods, non-prescription treatments, including more sexual activity, quitting smoking, pelvic-floor physiotherapy (PT), and lubricating agents or moisturizing cream [17]. While local phytoestrogens appear to exert a positive impact on VVA, improving maturation index, genital symptoms, vaginal pH level, morphology, and ER activation in the vaginal epithelium, in comparison to oral phytoestrogens, which are ineffective. Many menopausal symptoms, including VVA, are ameliorated by HRT, including estrogen progestins, tibolone, bazedoxifene, or purely estrogens in women without a uterus. The merely SERM recommended for the therapeutic management of VVA is ospemifene. It has a beneficial impact on the epithelium cells of the vagina while having little to no effect on the other estrogen-dependent organs. Pre-clinical tests on the breast have shown that it has an anti-estrogenic effect and appears to have no adverse impact on the endometrial and circulatory systems [18].

Prevention of osteoporosis: Women mainly use hormonal replacement therapy for an extended period to prevent complications like bone loss and postmenopausal fractures [15]. Reduced estrogen levels are the primary or only factor causing rapid bone turnover, decreased density of bone, lower bone strength, architectural destruction to the bone, and a greater possibility of fragility fractures. Treatment should be chosen based on an appropriate balance between expenditure, risks, and efficacy. In postmenopausal women, estrogen replacement treatment (ERT) can maintain and possibly raise BMD at all bone locations, including the femoral neck, lumbar spine, and forearm. Similarly, low-dose oral contraceptives (OCPs) can counteract the adverse effects of hypoestrogenism in young women [19].

Prevention of cardiovascular disease: The positive lipid regulation and estrogen's advantageous effects on the cells of the endothelium and vasculature are responsible for HT's protective effects on cardiovascular wellness [20]. The best cardiovascular disease risk evaluation for every woman is aided by considering genetic risk factors and non-traditional risk variables during her life. Transdermal MHT is favoured over oral treatment to lower the chance of VTE. Observational research indicates that transdermal female hormones may carry a lower risk of stroke and VTE than oral estrogens, and they also have less of an impact on biological markers of inflammation than oral estrogens. Additional rigorous lifestyle recommendations and treatment of concurrent heart disease risk factors are required in these potentially high-risk situations [21,22].

**Figure 2:**
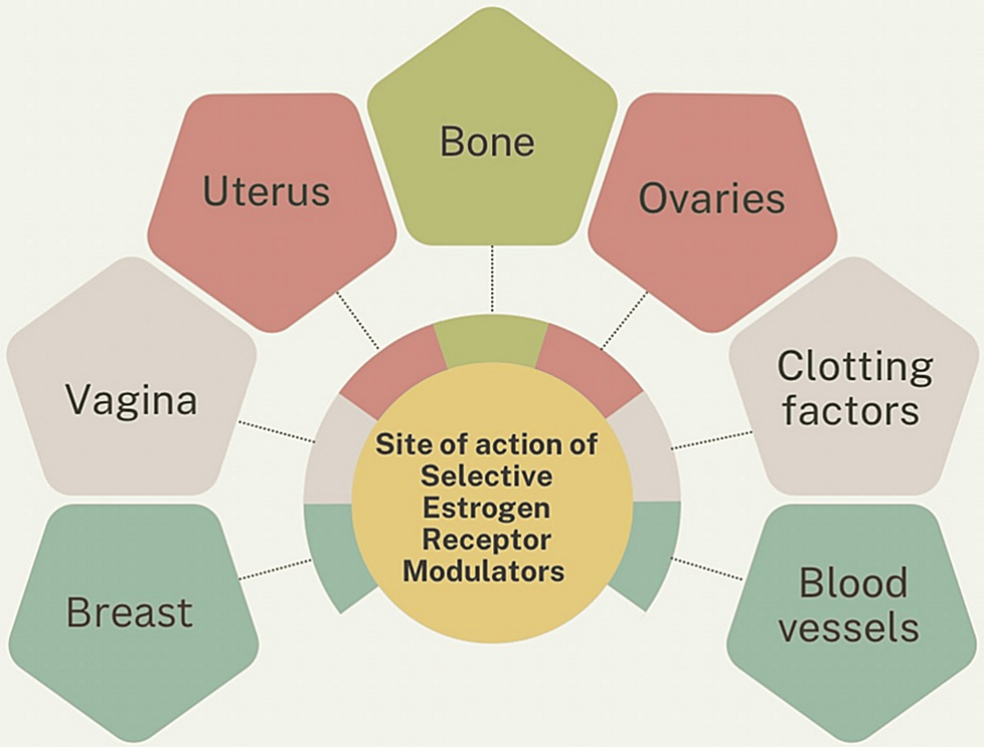
Various sites of action of selective estrogen receptor modulators (SERMs) Figure credit: Gunjan Motlani

SERMs are chemically distinct compounds that manage osteoporosis because they function as ER agonists in certain tissues of the target. However, they are also compounds of interest in breast cancer prevention because of their ability to act as ER antagonists in the tissues of the mammary glands, with specific impacts on the vagina and uterus that are contingent upon the way they interact with ER in these target organs. The “perfect” SERM continues to be searched after; it would have estrogen-like effects on serum lipids and bone, neutral effects on the womb, and antiestrogenic effects on breast tissue, but without any side effects linked to existing treatments [23]. Figure 2 enlists the sites of action of SERMs.

Tamoxifen: first generation SERM

The trans-isomer of a triphenylethylene substitute known as Tamoxifen was first identified while looking for anti-fertility drugs but was later shown to have anti-tumor potential. It is currently often used for managing breast cancer in every stage, and if administered with adjuvant therapy, it improves survival with little observable adverse effects [1,11]. The usage of Tamoxifen has been decreasing over time due to the emergence of selective ER degraders (SERDs) like fulvestrant and better aromatase inhibition drugs, and it is now primarily utilized as an adjuvant treatment for lower-risk premenstrual breast cancer and as a preventive treatment [24]. Lasofoxifene, ospemifene, and bazedoxifene are new SERM compounds that have been studied for their potential application in both the treatment and the prevention of osteoporosis. They are believed to be safer and more potent than earlier SERMs [23].

Raloxifene: second-generation SERM

Raloxifene, a nonsteroidal SERM, is employed for the treatment and prevention of osteoporotic fractures following menopause, as well as to lessen the incidence of carcinoma of the breast in postmenopausal women [25].

The SERM actions on various tissues of the female body are as follows:

1. On the uterus: The uterus is a crucial organ for clinical safety issues with continuous use of estrogenic drugs because the uterus is highly sensitive to estrogens. Regarding uterine stimulation, some SERMs differ from conventional estrogen [2,10].

2. On blood vessels: Hormonal replacement therapy raises the likelihood of venothrombotic events by around twofold. It is combined with risk factors such as surgery, thrombophilia, higher body mass index and immobilization. Venothrombotic events are more common when raloxifene is used [20].

3. Cognition: It is biologically conceivable that preventing cognitive deterioration and the emergence of dementia syndromes like Alzheimer's disease in postmenopausal women by keeping their levels of estrogen high by the use of ERT or estrogen and progesterone combination therapy (HRT). The effect appears to be mediated by several pathways, including dendritic sprouting, antioxidant effects, and impacts on different chemicals involved in cognitive functioning [26].

Newer SERMs 

Lasofoxifene: Clinical studies have shown that lasofoxifene dramatically enhanced bone mineral density (BMD) and reduced the levels of bone turnover indicators when compared to placebo. Also, it is linked to improved lipid profiles. It has also been shown that lasofoxifene lowers the incidence of vertebral and nonvertebral column fractures in postmenopausal women with osteoporosis and is connected to a lower risk of developing breast cancer that is ER-positive [27].

Bazedoxifene: The most appropriate SERM for osteoporosis treatment and prevention is the one that has agonistic effects on the bones while not activating uterine or breast tissues. In addition to minimizing bone loss, it would reduce the risk of breast soreness and endometrium stimulation, decreasing chances of endometrial growth, bleeding, polyp, hyperplasia, or even endometrium cancer. It has a beneficial role that lessens issues with vaginal dryness as it has agonistic activity at the vaginal level. Additionally, it has a favorable effect on lipid metabolism and vessel walls. Compared with raloxifene, the absence of vasomotor side effects was considered a benefit [28].

Arzoxifene: In menopausal women with a poor density of bone or osteoporosis, management with arzoxifene for four years dramatically decreased the incidence of vertebral fractures and invasive breast cancer. The likelihood of nonvertebral fractures was not reduced. Arzoxifene has no impact on cardiovascular diseases in women who are postmenopausal and have osteoporosis and is comparable to that of raloxifene. A higher incidence of venous thromboembolic events (VTE), endometrial malignancy, endometrial hyperplasia, many reports of pneumonia, upper respiratory infections (UTI), coughing, and severe unfavorable events of chronic obstructive pulmonary disease (COPD) were all linked to treatment with arzoxifene [29,30].

Ospemifene: The only and first nonhormonal medication, ospemifene, has been licensed by the FDA to treat moderately to severe painful intercourse resulting from vulvar and VVA, also known as atrophic vaginitis associated with menopause. The only SERM exhibiting nearly complete estrogen agonist effects on the epithelial cells of the vagina and neutral to minimal estrogenic influence on the lining of the endometrium is ospemifene. Ospemifene shows its tissue-specific impact on the breast, bone, uterus, coagulation markers, and serum lipid [31,32]. A summary of all the articles included in this review is listed in Table 1.

**Table 1:** Summary of the articles included in the review SERMs: Selective estrogen receptor modulator, HRT: Hormonal replacement therapy, BMD: Bone mineral density, CCR: Continuous combined regimen, HT: Hormonal therapy, ER: Estrogen receptor, GSM: Genitourinary syndrome of menopause, DHEAS: Dehydroepiandrosterone sulfate, VVA: Vulvovaginal atrophy, CVD: Cardiovascular diseases, pH: Potential of Hydrogen

Authors	Year	Findings
Burger et al. [1]	2000	An exciting discovery in the field of endocrine pharmacology is the emergence of tissue target-specific agents.
Bryant et al. [2]	2002	The SERMs are those substances that, depending on the type of tissue, exhibit estrogen agonist or antagonist activities.
Yasui et al. [3]	2001	Serum estradiol levels affect how HRT affects BMD, lipid profiles, and gonadotropin levels.
Andrews et al. [4]	1995	A CCR increases BMD more than a cyclical regimen of the same hormonal substances.
Kapoor et al. [5]	2021	HT for women with long-term medical illnesses such as diabetes, hypertension, dyslipidemia, obesity, autoimmune diseases, and venous thromboembolism.
Pollock et al. [6]	2022	The latest advances in the production of ER prodrugs are released through chemical reactions, light-mediated processes, and enzymatic reactions.
Katzenellenbogen et al. [7]	2000	SERMs ought to function according to ER subtypes.
Dowers et al. [8]	2006	The purpose of identifying potential new SERMs is to understand how the structures of SERMs affect the synthesis and reactivity of reactive compounds.
Martinkovich et al. [9]	2014	The mechanism of SERM specificity to tissue and demonstrates the therapeutic use of both established and novel SERMs.
Grese et al. [10]	1997	Structural variations between tamoxifen and raloxifene may influence the conformations of each drug's receptor/ligand complexes.
An et al. [11]	2016	SERMs are currently utilised for the treatment of osteoporosis, breast cancer and postmenopausal symptoms.
Pan et al. [12]	2022	HT for management of post-menopausal symptoms
Lethaby et al., [13]	2013	SERM decrease evening sweats and hot flashes in frequency or degree of severity in both perimenopausal and post-menopausal women
Shim et al. [14]	2021	Topical moisturizers, lubricants, systemic and local testosterone, oestrogens, intravaginal DHEAs, and SERMs can treat GSM.
Carroll et al. [15]	2002	Benefits, risks, and adverse effects associated with HRT
Naumova et al. [16]	2018	Diagnosis and treatment of VVA
Alvisi et al. [17]	2019	Signs, symptoms, diagnostic criteria, treatment, awareness related to VVA in postmenopausal women
Edwards et al. [18]	2016	Use of substances that is optimally balanced in terms of both PH and osmolality to treat vaginal dryness
Gambacciani et al. [19]	2013	SERMs such as Tamoxifen, Raloxifene, bazedoxifene and others are used in the prevention of osteoporosis. Comparison between them.
Mehta et al. [20]	2021	Types, routes, formulation, benefits, and side effects of HT.
Yang et al. [21]	2023	Sexual hormones have a protective role against CVD.
Sanchez-Borrego et al. [22]	2015	Efficacy and safety of HRT to control post-menopausal symptoms.
Maximov et al. [23]	2013	Actions of older and newer SERMs such as tamoxifen, raloxifene, ospemifene, lasofoxifene, bazedoxifene, and arzoxifene.
Howell et al. [24]	2023	Tamoxifen evolution.
Scott et al. [25]	1999	Raloxifene produces estrogen agonist activity on bone and lipid metabolism and estrogen antagonist activity on breast tissue and uterine endometrium.
Lethaby et al. [26]	2023	Molecular action of SERMs and its role in cognition.
Gennari et al. [27]	2007	Lasofoxifene has a role in the prevention and treatment of postmenopausal osteoporosis and also decreases ER+ breast cancer and vaginal atrophy.
Mitwally et al. [28]	2008	Bazedoxifene has been found to have a protective role for postmenopausal osteoporosis by reducing bone turnover.
Cummings et al. [29]	2011	Arzoxifene decreases invasive breast cancer and vertebral fractures
Jackson et al., [30]	2007	Arzoxifene has antagonistic activity on the endometrium and breast tissue and agonistic activity on lipid metabolism and bone with minimal side effects.
Soe et al. [31]	2023	Ospemifene is approved for the treatment of dyspareunia associated with vulvovaginal atrophy.
DeGregorio et al. [32]	2014	Ospemifene, its mechanism of action, and its action on various tissues.

## Conclusions

This article offers a thorough and perceptive summary of the changing function of SERMs in HRT. It emphasizes the importance of comprehending the intricate interactions between estrogen and its receptors in different tissues. It provides insight into the complex mechanisms through which SERMs can function as both agonists and antagonists. It also discusses the clinical uses of SERMs, such as the prevention of osteoporosis, the treatment of breast cancer, and the relief of postmenopausal symptoms. It also discusses the possible advantages of SERMs, including the treatment of VVA, the alleviation of vasomotor symptoms, and the avoidance of osteoporosis and cardiovascular disorders in women going through menopause. The article also discusses the continued development of novel SERMs, such as lasofoxifene, arzoxifene, bazedoxifene, and ospemifene, that offer better safety and efficacy. In summary, this article is a valuable source of information for researchers, clinicians, and anybody else curious about how women's health is changing. It offers an in-depth review of SERMs and all of their varied uses.
